# Thyroid Dysfunction, Vitamin B12, and Folic Acid Deficiencies Are Not Associated With Cognitive Impairment in Older Adults in Lima, Peru

**DOI:** 10.3389/fpubh.2021.676518

**Published:** 2021-09-06

**Authors:** Monica M. Diaz, Nilton Custodio, Rosa Montesinos, David Lira, Eder Herrera-Perez, Maritza Pintado-Caipa, Jose Cuenca-Alfaro, Carlos Gamboa, Serggio Lanata

**Affiliations:** ^1^Department of Neurology, University of North Carolina at Chapel Hill, Chapel Hill, NC, United States; ^2^Facultad de Salud Pública y Administración, Universidad Peruana Cayetano Heredia, Lima, Peru; ^3^Servicio de Neurología, Instituto Peruano de Neurociencias, Lima, Peru; ^4^Unidad de Diagnóstico de Deterioro Cognitivo y Prevención de Demencia, Instituto Peruano de Neurociencias, Lima, Peru; ^5^Unidad de Investigación, Instituto Peruano de Neurociencias, Lima, Peru; ^6^Servicio de Rehabilitación, Instituto Peruano de Neurociencias, Lima, Peru; ^7^Grupo de investigación Molident, Universidad San Ignacio de Loyola, Lima, Peru; ^8^Atlantic Fellow, Global Brain Health Institute, University of California, San Francisco, San Francisco, CA, United States; ^9^Servicio de Neuropsicología, Instituto Peruano de Neurociencias, Lima, Peru; ^10^Carrera de Psicología, Facultad de Ciencias de la Salud, Universidad Privada del Norte, Lima, Peru; ^11^Department of Neurology, University of California, San Francisco, San Francisco, CA, United States; ^12^Global Brain Health Institute, University of California, San Francisco, San Francisco, CA, United States

**Keywords:** dementia, metabolic disorder, thyroid dysfunction, vitamin B12, folic acid, Peru

## Abstract

**Background:** Reversible etiologies of cognitive impairment are common and treatable, yet the majority of mild cognitive impairment (MCI) and dementia research in Latin America has focused on irreversible, neurodegenerative etiologies.

**Objective:** We sought to determine if thyroid dysfunction and vitamin B12 and folate deficiencies are associated with cognitive disorders among older adults with memory complaints in Lima, Peru.

**Methods:** This was a retrospective review of patients who presented for cognitive evaluations to a multidisciplinary neurology clinic in Lima, Peru from January 2014 to February 2020. We included individuals aged ≥60 years, native Spanish-speakers, with at least a primary school educational level and a complete clinical assessment. Patients had either subjective cognitive decline (SCD), MCI, or dementia. One-way ANOVA and multiple logistic regression analyses were performed.

**Results:** We included 720 patients (330 SCD, 154 MCI, and 236 dementia); the dementia group was significantly older [mean age SCD 69.7 ± 4.1, dementia 72.4 ± 3.7 (*p* = 0.000)] and had lower folate levels than SCD patients. The MCI group had higher free T3 levels compared with SCD patients. Those with lower TSH had greater dementia risk (OR = 2.91, 95%CI: 1.15–6.86) but not MCI risk in unadjusted models. B12 deficiency or borderline B12 deficiency was present in 34% of the dementia group, yet no clear correlation was seen between neuropsychological test results and B12 levels in our study. There was no association between MCI or dementia and thyroid hormone, B12 nor folate levels in adjusted models.

**Conclusion:** Our findings do not support an association between metabolic and endocrine disorders and cognitive impairment in older Peruvians from Lima despite a high prevalence of B12 deficiency. Future work may determine if cognitive decline is associated with metabolic or endocrine changes in Latin America.

## Introduction

The risk of mild cognitive impairment (MCI) and dementia is known to increase exponentially with age (1–3). Worldwide, the number of people aged 65 years or older has risen from 6% in 1990 to 9% in 2019, and this figure is expected to double by the year 2050 (4). Following these demographic trends, the prevalence of dementia in Latin America (LA) is expected to rise to 27 million people by the year 2050 (5, 6).

Both reversible and irreversible etiologies of dementia and MCI exist (7, 8), yet the clinical evaluation of older adults with cognitive impairment in LA is often hindered by lack of access to skilled clinicians with resources and training needed to diagnose dementia appropriately and a limited consensus on the best approaches for evaluation and diagnosis (9). Further adding to this diagnostic challenge, the medical literature on dementia in LA has mostly focused on irreversible, neurodegenerative etiologies such as Alzheimer's disease (AD), whereas relatively little emphasis has been placed on reversible or treatable etiologies, especially metabolic and endocrine disorders known to cause cognitive impairment (10–13).

Guidelines published by the American Academy of Neurology (AAN) recommend screening for certain metabolic and endocrine disorders, such as B12 and folic acid (or folate) deficiencies and thyroid dysfunction, when evaluating a person with cognitive impairment (14). The prevalence of these and other potentially reversible causes of dementia is as high as nearly 20% in one study of patients recently diagnosed with dementia from Brazil (15); thus, screening for these relatively common and potentially treatable conditions in LA may be of value, particularly in regions with poor nutritional status and lack of mandatory vitamin fortification. Serum laboratory analyses of potentially reversible etiologies of dementia, such as thyroid hormone, vitamin B12 and folate levels are cost-effective, highly-accessible and may identify potentially treatable etiologies of cognitive impairment in LA (15–17).

Vitamin B12 deficiency is common in older adults and potentially treatable (18). Particularly among those with MCI and dementia, low levels of vitamin B12 may worsen cognition among older adults with ApoE4 allele or with depression (19). Additionally, folate deficiency, arising from insufficient dietary folate or gut malabsorption, leading to high serum levels of the amino acid homocysteine, has been linked to dementia (11, 20, 21). One systematic review found that a folate supplemented diet led to improved cognition in mouse models (21), however, another review found that folic acid supplementation did not improve cognition in older adults (22). Thus, the role of folate in improving cognition is conflicting. Identifying folate or vitamin B12 deficiency and homocysteine levels in patients with a major neurocognitive disorder, such as dementia, may help reverse or improve the cognitive impairment associated with these conditions. Thyroid hormone dysfunction, particularly clinical hyperthyroidism and chronic hypothyroidism, is also associated with increased dementia risk (23, 24), yet in LA, few studies have investigated this relationship (25, 26) and no study has investigated this relationship in Peru. Therefore, metabolic and endocrine disorders, such as thyroid hormone and vitamin deficiencies are important to consider when evaluating both (i) persons without an existing dementia diagnosis presenting for an initial consultation for cognitive complaints, and (ii) older adults who have a diagnosis of dementia, given medical management of these hormonal or metabolic alterations may help reverse or prevent further cognitive decline (27).

To our knowledge, no research studies have explored associations between metabolic and endocrine disorders and cognitive impairment in Peru, and only a few studies have explored these associations in other countries in the region. Therefore, we present a retrospective, cross-sectional study characterizing serum levels of thyroid hormone, vitamin B12 and folate among older adults presenting to a multidisciplinary neurology clinic in Lima, Peru for an initial consultation for cognitive complaints, diagnosed as either subjective cognitive decline, having MCI, or dementia. We hypothesize that metabolic and endocrine disorders are associated with cognitive impairment in older adults living in Lima, Peru.

## Methods

### Study Design

We conducted a retrospective review of medical records of all patients who presented for an initial consultation for a cognitive complaint to a multidisciplinary neurology clinic of the Instituto Peruano de Neurociencias (IPN) in Lima, Peru from January 2014 to February 2020. The protocol was approved by the institutional review board of the Hospital Nacional Docente Madre Niño San Bartolomé.

### Participants

All patients evaluated at IPN undergo a standard evaluation that includes data collection of demographic (age, sex, and years of education), clinical (including functional evaluation), and neurological characteristics and findings, cognitive and a complete neuropsychiatric evaluation, followed by serum laboratory analyses and neuroimaging.

#### Inclusion Criteria

We included individuals 60 years of age or older who were native Spanish-speakers, completed at least 6 years of primary school education and who had a complete clinical assessment (demographic data; clinical, neurological, cognitive, neuropsychiatric evaluations and testing; serum laboratory analyses and neuroimaging).

#### Exclusion Criteria

**Figure 1:**
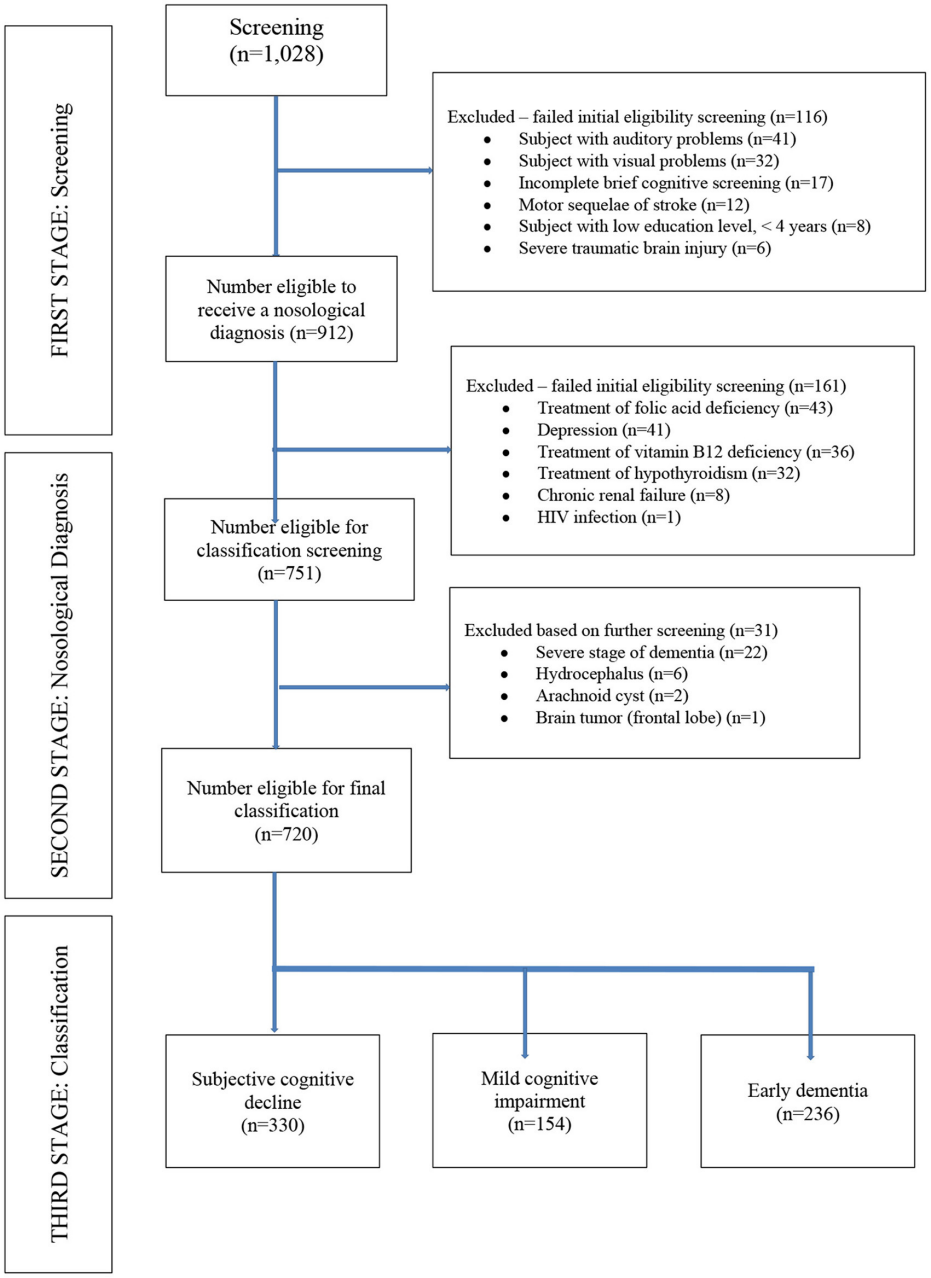
Flowchart of participants included in the study by study phase (*N* = 720).

We excluded patients with an educational levels <4 years and those with a history of substance abuse or addiction, chronic recurrent depression, chronic renal failure, HIV infection, neurological sequelae of severe traumatic brain injury, as well as any medical condition that could affect their performance on cognitive testing (auditory or visual difficulties, severe dementia impeding ability to complete cognitive testing, hydrocephalus, arachnoid cyst, brain tumors, motor sequelae of cerebrovascular disorders or traumatic sequelae). We also excluded those with who did not complete brief cognitive screening. We also excluded patients receiving treatment for thyroid dysfunction and vitamin B12 or folic acid supplementation at the time of study entry. All patients with a period of longer than 30 days between the first and second phases of the clinical evaluations were also excluded (Figure 1).

#### Study Procedures

Clinical evaluations of patients presenting to our clinics for evaluation of cognitive complaints are performed in three successive consecutive phases as follows: (1) screening phase to determine which patients are cognitively-impaired; (2) determination of the etiology of cognitive impairment; and (3) final classification of the subtype of disease. In the screening phase, data from a structured clinical interview and clinical examination (including functional independence) were collected to determine general demographic and clinical characteristics, medication history at the time of assessment, and past medical and mental health history. Medications known to have an effect on cognition were collected, including opioid analgesics, decongestants, anti-spasmodics, anti-emetics, anti-cholinergics, anti-arrhythmics, anti-depressants, anti-psychotics, anti-anxiety, or anti-epileptic medications. Due to the effect of vascular or depressive risk factors on thyroid hormone levels (28), as well as cognitive impairment and dementia (29), we collected the following data: anthropometric data (weight, height, waist and hip circumference), number of depressive symptoms, self-reported history of transient ischemic attack (TIA)/stroke, heart disease (self-reported history of myocardial infarction, atrial fibrillation, digitalis use, or angina pectoris), hypertension (self-reported history, or diastolic blood pressure [DBP] ≥ 95 mmHg and systolic blood pressure [SBP] ≥ 160 mmHg at study visit), diabetes mellitus (self-reported or use of diabetic medication) and physical activity level [defining regular exercise as that which induces frequent sweating at least 1 day per week, as previously described (30)].

In the first phase (screening phase), we applied the Pfeffer Functional Activities Questionnaire (PFAQ) (31) and two brief cognitive screening tests, the Mini Mental State Examination (MMSE) (32) and the INECO Frontal Screening (IFS) exam (33). MMSE and IFS were administered to all study subjects; while PFAQ was administered to the caregivers/chaperones accompanying each patient to the clinic visit. In the second phase, patients were evaluated using serum laboratory tests (vitamin B12, folic acid, free T3 [fT3] and free T4 [fT4], and ultra-sensitive Thyroid Stimulating Hormone [TSH] levels), and neuroimaging (CT scan or MRI of the brain). In this phase, all patients also underwent a complete neuropsychological test battery administered by a licensed neuropsychologist. The neuropsychological battery included the following tests: Rey Auditory Verbal Learning Test, Logical Memory Subtest of Weschler Memory Scale-Revised, Trail Making Test A and B, Rey-Osterrieth Complex Figure Test, Boston Naming Test, Wisconsin Card Sorting Test, Letter-Number (subtest of the Weschler Adult Intelligent Scale-III), Digit Span and Clinical Dementia Rating (CDR) scale, as has previously been described (34). Neuropsychiatric symptoms were assessed by means of the Neuropsychiatric Inventory (NPI). We used NPI-12, a clinical informant interview surveying the following behavioral disturbances: delusions, hallucinations, agitation/aggression, irritability, depression, anxiety, euphoria, disinhibition, aberrant motor behavior, apathy, sleep, and appetite and was administered by two trained professionals. With a maximum of 144 points, the NPI-12 delivers a total symptom score based on frequency and severity of each subdomain. According to the criteria-based rating scheme, the severity of each manifestation was classified into four grades (from 1 to 3; 0 if absent), and the frequency of each manifestation was also classified into five grades (from 1 to 4; 0 if absent). The NPI score (severity x frequency) was calculated for each manifestation (range of possible scores: 0–12). The presence of a symptom was expressed as an NPI subset score >0 (35).

Finally, in the third phase, a diagnosis of MCI or dementia was made based on clinical impression and the results of the complete neuropsychological evaluation, blood tests and neuroimaging, based on previously published criteria for diagnosing major and minor neurocognitive disorders in the Diagnostic and Statistical Manual of Mental Disorders-5 (DSM-5) (36).

All assessments in the first phase were administered by a study neurologist or geriatrician who was blinded to the neuropsychological testing results that were administered by the neuropsychologists. Diagnostic classification disagreements among the evaluators was resolved by consensus among the study team members comprised of neurologists, geriatricians and neuropsychologists. Individuals who presented with cognitive complaints but had normal results on all brief cognitive tests and had normal scores on the PFAQ and CDR were classified as having subjective cognitive decline (SCD) (37, 38). Thus, all participants included were assigned to one of three groups based on the above evaluations and clinical consensus: SCD, MCI or dementia group. For those who had dementia, we further characterized them by sub-type of dementia including: (1) vascular dementia [defined as a cognitive syndrome caused by vascular cognitive impairment due to cerebrovascular disease with manifestations of cognitive impairment exceeding those observed in normal aging; vascular dementia is the final stage of vascular cognitive impairment (39)]; (2) frontotemporal dementia (40); (3) mixed dementia [requires the existence of a typical AD and dementia related to cerebrovascular disease, as previously described (41, 42)]; (4) Dementia with Lewy bodies (43); or (5) AD (utilizing criteria published by McKhann et al. (44), by evidence of progressive cognitive decline on serial evaluations based on information from informants and cognitive testing by either formal neuropsychological evaluation (34).

### Cognitive and Functional Assessments

The brief cognitive screening tests applied in this study included the Peruvian Spanish adaptation of the MMSE and the validated Spanish version of the IFS. The MMSE and IFS were selected given their utility among persons with educational levels of at least primary school and have been used widely among Spanish-speaking older adults (45). Moreover, the Peruvian Spanish version of the MMSE has been found to be highly sensitive and specific when comparing dementia vs. SCD (sensitivity 91%, specificity 75%) and dementia vs. MCI (sensitivity 87%, specificity 75%) among Peruvians living in Lima, Peru (46, 47). The MMSE is a brief cognitive screening tool that evaluates orientation (in time and space), immediate recall (or 3-word recall), attention and calculation, delayed recall, language (naming, repetition, reading, writing, performing verbal commands) and constructive praxis. We used the Peruvian version, modified from the Buenos Aires, Argentina version (32), and is administered in about 10 min, on average, and is based on a maximum score of 30 with a score <26 indicating cognitive impairment in a Peruvian population with >7 years of education (48) (Supplementary Material 1). The IFS is a screening test that uses eight sub-tests to assess executive function, with a maximum of 30 points, including motor programming (3 points), conflicting instructions (3 points), motor inhibitory control (3 points), backward digit span (6 points), verbal working memory (2 points), spatial working memory (4 points), abstraction capacity (3 points), verbal inhibitory control (6 points), where lower scores indicate poorer cognitive performance. The maximum score on the IFS is 30, and a score <23 indicates cognitive impairment in a Peruvian population with >10 years of education (33) (Supplementary Material 2). Functional assessment was completed using the PFAQ, which includes 11 questions assessing activities of daily living (ADLs), including an additional question on ability of the patient to take their own medications correctly. The maximum score on the PFAQ is 33, and a score >7 indicates functional impairment (49) (Supplementary Material 3).

### Laboratory Analyses

A blood sample (3–5 ml of blood) was collected intravenously from the upper limb of the participants who were fasting for at least 12 h. Serum levels of folic acid < 3 ng/ dL and vitamin B12 <80 pg/mL were considered deficient. A low cut-off of < 80 pg/mL, previously utilized in other published studies (50, 51), was selected to ensure that any relationships detected between vitamin B12 levels and cognitive status were accurate. Of note, homocysteine and methylmalonic acid levels are unavailable in the laboratory where these laboratory results were obtained. Thyroid dysfunction was evaluated with measurements of levels of TSH, fT3, and fT4. According to verified laboratory reference ranges, the normal serum levels of TSH, fT3 and fT4 were 0.55–4.78 mIU/l, 3.50–6.50 pmol/l, and 11.50–22.70 pmol/l, respectively. Laboratory cut-offs for hypothyroidism were TSH level > 4.78 mIU/l, fT4 < 11.50 or fT3 < 3.50 pmol/l, and hyperthyroidism were TSH level <0.55 mIU/l, fT4 > 22.70 or fT3 > 6.50 pmol/l. Based on thyroid hormone levels, patients were classified into four categories: subclinical hyperthyroidism (low serum TSH with normal levels of fT3 and fT4), euthyroidism (TSH, fT3, and fT4 at normal values), subclinical hypothyroidism (elevated serum TSH with normal levels of fT3 and fT4) and clinical hypothyroidism (elevated serum TSH with low levels of fT3 and/or fT4), based on previously published criteria (52).

### Statistical Analysis

Descriptive statistics were performed comparing demographics, brief cognitive screening and laboratory results of each cognitive group against one another (subjective cognitive decline-MCI, subjective cognitive decline-dementia, MCI-dementia) applying Chi-square (for categorical variables) or Analysis of One-Way Variance (ANOVA) for continuous variables. Bonferroni corrections were applied to adjust for these multiple comparisons. Participants were also divided into five quintiles based on lowest to highest TSH levels to allow for a logistic regression analysis to be performed using the fifth (or highest) quintile as the reference group. Logistic regression was used to assess the association of thyroid dysfunction with MCI and dementia (univariable logistic regression analyses were performed and multivariable logistic regression analyses adjusted for age, sex and BMI). Linear regression models comparing thyroid function, vitamin B12 and folate levels to MMSE and IFS scores, adjusted for age, sex, years of education and body mass index, were completed. For analyses in which vitamin B12 and folate levels were the dependent variables, conditional multiple logistic regression analyses were applied to obtain the odds ratios (OR) and *p*-value for any trends in the models. The first model was a crude model without variable adjustment. In the second model (adjusted model) the analyses were adjusted for regular exercise [utilized as a marker of cardiovascular health, a known risk factor for cognitive impairment (29)]. In the third model, where Vitamin B12 level was the dependent variable, any value >2,000 pg/mL was considered an outlier and excluded from the model. We also completed a sub-analysis of exploring the effect of folate and Vitamin B12 levels on AD. All calculated *P*-values were unpaired and two-tailed with differences considered significant at *p* < 0.05. Data were evaluated using 95% confidence intervals using STATA software (version 12.0).

## Results

Per the study protocol, we reviewed the electronic medical record system of IPN and found 1,028 patients eligible for screening from which 720 clinical records were obtained between January 2014 to February 2020 that met inclusion criteria (Figure 1). Of these, 330 patients were diagnosed with SCD, 154 patients with MCI and 236 patients with dementia (146 AD, 45 vascular dementia, 18 mixed dementia, 10 dementia associated with Parkinson's Disease, 10 behavioral variant frontotemporal dementia, 4 primary progressive aphasia, and 3 dementia with Lewy bodies). Of the entire sample, vitamin B12 deficiency was present in 21% (*n* = 151), thyroid dysfunction in 7% (*n* = 50) and folic acid deficiency in 1.7% (*n* = 12). Other risk factors for cognitive impairment included medications known to have an effect on cognition in 9% (*n* = 64), hepatitis B/C in 3% (*n* = 21), any history of traumatic brain injury in 2% (*n* = 14). The MCI and dementia groups were each older compared with the subjective cognitive decline (SCD) group, with no differences in sex or educational level between the groups. Mean ± SD of thyroid hormone levels (fT4, fT3, and TSH), vitamin B12 and folate levels were within normal range for all three cognitive groups (Table 1). Both MMSE and IFS scores were lower in the dementia and MCI groups, compared with the SCD group (*p* = 0.000 for both comparisons; Table 1). The mean PFAQ score for the dementia group was 15.19 ± 2.63; the mean CDR score for the dementia group was 1.58 ± 0.69, and the mean score for the NPI was 13.8 ± 4.37 in the dementia group; all were significantly greater in the dementia group compared with both the MCI and SCD groups individually (Table 1).

**Table 1:** Demographic, cognitive, and metabolic characteristics of the sample by cognitive stage^**^.

**Characteristics**	**SCD** **(*n* = 330)**	**MCI** **(*n* = 154)**	**Dementia** **(*n* = 236)**	***P*-value** **(SCD vs. MCI)**	***P*-value** **(MCI vs. D)**	***P*-value (SCD vs. D)**
**Age, years (mean ± SD)**	69.65 ± 4.13	70.26 ± 3.51	72.42 ± 3.66	0.416	**0.021**	**0.000**
**Female, n (%)**	171 (51.8%)	74 (48%)	121 (51.3%)	0.078	0.129	0.563
**Education, years (mean ± SD)**	12.71 ± 3.11	11.68 ± 3.16	11.16 ± 2.27	0.087	0.217	0.061
**BMI, kg/m** ^**2**^ **(mean ± SD)**	22.59 ± 6.5	23.2 ± 5.4	22.48 ± 4.9	0.142	0.235	0.636
**MMSE, score (mean ± SD)**	25.61 ± 1.5	22.18 ± 2.1	17.79 ± 2.2	0.000^*^	0.000^*^	0.000^*^
**IFS, score (mean ± SD)**	26.73 ± 1.3	21.12 ± 1.2	15.12 ± 1.9	0.000^*^	0.000^*^	0.000^*^
**PFAQ, score (mean ± SD)**	2.11 ± 1.33	4.21 ± 1.18	15.19 ± 2.63	**0.003**	0.000^*^	0.000^*^
**CDR, score (mean ± SD)**	0.47 ± 0.19	0.81 ± 0.27	1.58 ± 0.69	**0.015**	**0.011**	**0.002**
**NPI, score (mean ± SD)**	7.1 ± 0.29	8.2 ± 1.41	13.8 ± 4.37	**0.001**	**0.003**	0.000^*^
**B12 pg/ml (mean ± SD)**	364.25 ± 65.4	404.20 ± 123.6	356.46 ± 36.4	0.162	0.092	0.813
**Folic Acid ng/dl (mean ± SD)**	9.3 ± 4.4	9.4 ± 3.7	8.2 ± 2.8	0.621	0.072	**0.024**
**Free T3 pmol/L (mean ± SD)**	4.4 ± 0.6	4.7 ± 0.6	4.4 ± 0.3	**0.032**	0.117	0.472
**Free T4 pmol/L (mean ± SD)**	15.3 ± 1.2	15.1 ± 0.9	14.9 ± 2.2	0.296	0.158	0.172
**TSH mU/L (mean ± SD)**	2.0 ± 1.0	2.1 ± 1.3	1.9 ± 1.3	0.359	0.084	0.168

*BMI, body mass index; CDR, Clinical Dementia Rating scale; D, dementia; IFS, INECO Frontal Screening; NPI, Neuropsychological Inventory; MCI, mild cognitive impairment; MMSE, Mini Mental State Examination; PFAQ, Pfeffer Activities of Daily Living Questionnaire; SCD, subjective cognitive decline*.

* *p < 0.001*.

*Instituto Peruano de Neurociencias, 2014–2020*.

** *One-way ANOVA (continuous variables) or Chi-square (categorical variables) were performed*.

*The bold values mean that the p-value < 0.05*.

### Comparative Analyses Between Serum Thyroid Hormone With Demographic and Cognitive Characteristics

Of 720 participants in this study, 670 (93%) had normal thyroid function, 18 (2.5%) had hypothyroidism, 23 (3.2%) had subclinical hypothyroidism, and 9 (1.3%) had subclinical hyperthyroidism (Table 2). Higher levels of fT3 were observed among the MCI group compared with the SCD group (*p* = 0.032), but not among the dementia group compared with the SCD group (*p* = 0.472). There were no statistically significant differences in fT4 or TSH levels between the groups (Table 1). No statistically significant association was found between serum thyroid hormone levels, age, BMI, years of education and cognitive test results in the MCI group (Supplementary Table 4). However, fT3 levels were inversely associated with age in the dementia group (γ = −0.31, *p* < 0.05; Supplementary Table 4). Serum TSH levels were inversely associated with MMSE and IFS scores in the dementia group (γ = −0.21, *p* < 0.05; γ = −0.32, and *p* < 0.05, respectively; Table 3), but no other significant associations in the MCI and SCD groups (Table 3). There were no significant associations between fT3 and fT4 levels and dementia, MCI nor SCD independently (Table 3). Compared with the fifth (Q5, or highest) quintile, participants in the lowest (Q1) and second (Q2) lowest TSH quintiles had greater risk of dementia (Table 4), but no statistically significant associations were noted between TSH quintiles and MCI (Table 4) nor SCD risk (Table 4). fT4 levels were inversely associated with years of education in the SCD group (γ = −0.35, *p* < 0.05; Supplementary Table 4).

**Table 2:** Demographic, clinical, and cognitive characteristics of participants with normal thyroid function and thyroid dysfunction.

**Characteristic**	**Hypothyroidism** **(*n* = 18)**	**Subclinical hypothyroidism** **(*n* = 23)**	**Normal thyroid function** **(*n* = 670)**	**Subclinical hyperthyroidism** **(*n* = 9)**	*P*-value^*^
**SCD**, ***n*****(%)**	5 (27.8%)	5 (21.7%)	176 (26.3%)	1 (11.1)	0.875
**MCI**, ***n*****(%)**	3 (16.7%)	5 (21.7%)	145 (21.6%)	5 (55.5%)	0.956
**Dementia**, ***n*****(%)**	10 (55.5%)	13 (56.5%)	349 (52.1%)	3 (33.3%)	0.862
**Age, years (mean ± SD)**	65.6 ± 8.8	63.4 ± 9.1	62.2 ± 8.3	64.1 ± 8.9	0.068
**Education, years (mean ± SD)**	11.7 ± 3.5	10.8 ± 3.6	11.1 ± 2.9	10.2 ± 2.9	0.456
**Female**, ***n*****(%)**	9 (50%)	13 (56.5%)	352 (52.5%)	5 (55.5%)	0.403
**BMI, kg/m** ^**2**^ **(mean ± SD)**	23.1 ± 2.3	23.2 ± 3.1	22.9 ± 2.9	23.3 ± 2.6	0.856
**MMSE score (mean ± SD)**	21.3 ± 4.5	20.8 ± 8.1	20.9 ± 7.6	22.1 ± 5.1	0.435
**IFS score (mean ± SD)**	22.7 ± 2.3	20.2 ± 1.9	23.1 ± 1.3	20.8 ± 2.2	0.135

*BMI, body mass index; IFS, INECO frontal screening; MCI, mild cognitive impairment; SCD, subjective cognitive decline; MMSE, Mini Mental State Examination*.

* *One-way ANOVA (continuous variables) or Chi-Square (categorical variables) performed between normal thyroid function and thyroid dysfunction groups (hypothyroidism + subclinical hypothyroidism + subclinical hyperthyroidism)*.

*Instituto Peruano de Neurociencias, 2014–2020*.

**Table 3:** Comparison of free T3, free T4, TSH, vitamin B12, and folic acid with scores on brief cognitive tests^*^ among patients with dementia, mild cognitive impairment and subjective cognitive decline.

**Variable**	**Dementia group**						**MCI group**						**SCD group**					
	**MMSE**			**IFS**			**MMSE**			**IFS**			**MMSE**			**IFS**		
	**β (SE)**	***P*-value**	**95% CI for β**	**β (SE)**	***P*-value**	**95% CI for β**	**β (SE)**	***P*-value**	**95% CI for β**	**β (SE)**	***P*-value**	**95% CI for β**	**β (SE)**	***P*-value**	**95% CI for β**	**β (SE)**	***P*-value**	**95% CI for β**
Free T3^**^	−0.23 (0.19)	0.072	−0.65 to 0.35	−0.15 (0.88)	0.089	−0.56 to 0.29	−0.09 (0.23)	0.085	−0.03 to 0.11	−0.06	0.082	−0.02 to 0.21	0.17 (0.61)	0.071	0.06 to 0.31	0.11 (0.29)	0.091	0.07 to 0.57
Free T4	−0.15 (0.37)	0.145	−0.46 to 0.25	−0.21 (0.71)	0.092	−0.43 to 0.16	−0.10	0.071	−0.07 to 0.21	−0.10	0.127	−0.06 to 0.35	−0.06 (0.81)	0.164	0.02 to 0.24	−0.21 (0.17)	0.252	0.07 to 0.49
TSH	−0.32 (0.24)	**0.010**	−1.49 to 0.16	−0.37 (0.97)	**0.004**	−3.26 to 0.73	−0.37 (0.19)	0.081	−0.92 to 0.58	−0.32 (0.72)	0.086	−0.13 to 0.62	−0.27 (0.19)	0.092	−1.49 to 0.52	−0.23 (0.48)	0.082	−1.26 to 0.49
Vitamin B12	0.03 (0.16)	0.062	0.01 to 0.21	0.09 (0.32)	0.074	0.05 to 0.42	0.07 (0.22)	0.095	0.02 to 0.15	0.10 (0.27)	0.146	0.04 to 0.33	0.12 (0.23)	0.213	0.04 to 0.69	0.19 (0.61)	0.084	0.06 to 0.61
Folic acid	0.05 (0.38)	0.060	0.03 to 0.52	0.12 (0.21)	0.082	0.07 to 0.34	0.08 (0.44)	0.083	0.02 to 0.41	0.21 (0.19)	0.077	0.09 to 0.42	0.13 (0.56)	0.087	0.07 to 0.46	0.21 (0.49)	0.273	0.09 to 0.49

*IFS, INECO Frontal Screening; MCI, mild cognitive impairment; MMSE, Mini Mental State Examination; SE, standard error; SCD, subjective cognitive decline; TSH, thyroid stimulating hormone*.

*Linearity test: Pearson correlation coefficient, r = 0.8; p = 0.033; coefficient of determination, R^2^ = 0.66*.

* *Linear regression models adjusted for age, sex, years of education, and body mass index*.

** *Normal serum free T3, 3.50–6.50 pmol/l; normal serum free T4 11.50–22.70 pmol/l; normal serum TSH, 0.55–4.78 mIU/l; vitamin B12 deficiency <80 pg/mL; normal folic acid levels, ≥3 ng/ dL*.

*Instituto Peruano de Neurociencias, 2014–2020*.

*The bold values mean that the p-value < 0.05*.

**Table 4:** Regression analyses^*^ of serum TSH levels (in quintiles) and risk of dementia, mild cognitive impairment, and subjective cognitive decline.

Variable^**^	**Dementia**				**MCI**				**SCD**			
	**β (SE)**	***P*-value**	**OR (β)**	**95% CI for OR (β)**	**β (SE)**	***P*-value**	**OR (β)**	**95% CI for OR (β)**	**β (SE)**	***P*-value**	**OR (β)**	**95% CI for OR (β)**
TSH (1)	1.03 (0.46)	**0.021**	2.91	1.15–6.86	0.81	0.069	1.47	(0.83–3.21)	0.66 (0.41)	0.128	1.39	0.59–4.18
TSH (2)	0.89 (0.37)	**0.032**	2.63	1.09–6.31	0.63	0.072	1.23	(0.56–3.41)	0.59 (0.71)	0.073	1.33	0.51–4.93
TSH (3)	0.56 (0.37)	0.271	1.62	0.60–3.87	0.61	0.456	1.12	(0.82–4.51)	0.49 (0.37)	0.124	1.28	0.46–4.17
TSH (4)	0.45 (0.42)	0.319	1.56	0.65–2.97	0.26	0.092	1.83	(0.74–3.47)	0.51 (0.36)	0.092	1.15	0.53 to 3.71

*β, beta; OR, odds ratio; CI, confidence interval; SE, standard error; TSH, thyroid stimulating hormone*.

*Linearity test: Pearsons correlation coefficient, r = 0.7; p = 0.016; coefficient of determination, R^2^ = 0.74*.

* *Logistic regression models adjusted for age, sex, years of education, and body mass index*.

** *Fifth quintile is the reference group*.

*Quintiles: Q1: 0.26–1.12 mIU/L; 1.13–1.33 mIU/L; Q3: 1.34–1.86 mIU/L; Q4: 1.87–4.25 mIU/L; Q5: 4.26–28.5 mIU/L*.

*Instituto Peruano de Neurociencias, 2014–2020*.

*The bold values mean that the p-value < 0.05*.

Compared with the normal thyroid function group, we found no significant association between MCI and thyroid dysfunction both in the univariate analysis (Table 2) and in the multivariate analysis after adjustment for age, sex, years of education, and BMI (OR = 0.71, 95% CI: 0.11–4.36; OR = 1.13, 95% CI: 0.24–7.92; OR = 0.37, 95% CI: 0.07–1.28, respectively). Similarly, we found no association between thyroid dysfunction and dementia in the multivariate model (OR = 1.31, 95% CI: 0.39–4.51; OR = 1.18, 95% CI: 0.12–6.43; OR = 1.13, 95 % CI: 0.13–5.36, respectively).

### Comparative Analyses Between Serum Folate and Vitamin B12 Levels With Demographic and Cognitive Characteristics

Of all participants, 151 had vitamin B12 deficiency (28% with SCD, 32.5% with MCI, 49% of these with dementia), however there were no statistically significant differences between those in the vitamin B12 deficiency group and those in the normal B12 group in any of the groups (Table 5). A total of 12 participants had folic acid deficiency in the cohort (25% SCD, 42% MCI, and 33% dementia). Given the small number of participants with folic acid deficiencies, comparisons between groups were not possible.

**Table 5:** Demographic, clinical, and cognitive characteristics of participants with normal levels of vitamin B12 and vitamin B12 deficiency^*^.

**Characteristic**	**Vitamin B12 deficiency** **(*N* = 151)**	**Intermediate Vitamin B12 status** **(*N* = 96)**	**Vitamin B12 normal** **(*N* = 473)**	P^**^	P^***^
**SCD**, ***n*****(%)**	28 (18.5%)	15 (15.6%)	236 (49.9%)	0.079	0.064
**MCI**, ***n*****(%)**	49 (32.5%)	37 (38.5%)	156 (32.9%)	0.863	0.091
**Dementia**, ***n*****(%)**	74 (49%)	44 (45.8%)	81 (17.2%)	0.066	0.072
**Age, years (mean ± SD)**	64.8 ± 7.2	66.3 ± 6.4	65.4 ± 5.1	0.143	0.317
**Education, years (mean ± SD)**	10.5 ± 3.2	10.9 ± 2.9	11.3 ± 2.9	0.633	0.734
**Female**, ***n*****(%)**	78 (51.7%)	52 (54.2%)	239 (50.5%)	0.625	0.691
**BMI, kg/m** ^**2**^ **(mean ± SD)**	22.5 ± 3.1	22.9 ± 2.7	23.4 ± 3.2	0.726	0.811
**MMSE score (mean ± SD)**	21.5 ± 4.3	23.4 ± 3.9	21.2 ± 5.6	0.628	0.158
**IFS score (mean ± SD)**	22.9 ± 2.1	21.6 ± 3.3	20.6 ± 3.1	0.319	0.097

*BMI, body mass index; IFS, INECO Frontal Screening; MCI, mild cognitive impairment; MMSE, Mini Mental State Examination; SCD, subjective cognitive decline*.

* *Vitamin B12 deficiency (<80 pg/mL); indeterminate Vitamin B12 status (81–200 pg/mL); normal Vitamin B12 (>200 pg/mL)*.

** *One-way ANOVA (continuous variables) or Chi-square (categorical variables) for normal vitamin B12 group vs vitamin B12 deficiency group*.

*** *One-way ANOVA (continuous variables) or Chi-square (categorical variables) for normal vitamin B12 group vs. intermediate vitamin B12 group*.

*Instituto Peruano de Neurociencias, 2014–2020*.

Lower serum folate concentrations were observed in the dementia group compared to those who had SCD (*p* = 0.024), but no differences in Vitamin B12 levels were observed between the groups (Table 1). Serum B12 and folate levels were each inversely associated with age in the dementia group (γ = −0.35, *p* < 0.05; γ = −0.43, *p* < 0.05, respectively; Supplementary Table 5). There were no statistically significant associations between age, BMI, years of education and vitamin B12 nor folic acid levels within the MCI and SCD groups (Supplementary Table 5). No significant associations were observed between serum vitamin B12 and folate levels with MMSE and IFS scores (Table 3; Supplementary Table 5). When stratifying vitamin B12 levels by three groups (deficient, <80 pg/mL; indeterminate, 81–200 pg/mL; normal, >200 pg/mL) we found that there were no significant differences in vitamin B12 status in the MCI group, but vitamin B12 deficiency and indeterminate levels of Vitamin B12 were both associated with dementia compared with normal B12 levels. Most patients had normal vitamin B12 levels in the SCD group and more patients in the dementia group had vitamin B12 deficiency (Supplementary Table 6). We completed a sub-analysis comparing the effect of folate levels and vitamin B12 levels on having a diagnosis of AD (*n* = 146) vs. SCD and found no significant effect of either folic acid levels or vitamin B12 levels stratified by quintiles in adjusted models; data not shown. Serum folate concentrations were inversely associated with cognitive impairment in the unadjusted model when comparing 5th vs. 1st quintiles. However, after additional adjustment for regular exercise, this association was no longer significant (Table 6).

**Table 6:** Odds ratio (OR) for the prevalence of cognitive impairment by quintiles of serum folic acid and vitamin B12 concentrations. Instituto Peruano de Neurociencias, 2014–2020.

Variable^*^	**MCI + D**	**SCD**	**Prevalence of cognitive impairment**			
			**Crude model**		**Adjusted model** ^**b**^	
	***N* = 390**	***N* = 330**	**OR** **(95% CI)**	***P*-value^**a**^**	**OR** **(95% CI)**	***P*-value^**a**^**
**Folic acid (ng/dL)**				**0.021**		0.098
**Q1**	128	56	1.00 (reference)		1.00 (reference)	
**Q2**	90	89	0.49 (0.21–1.02)		0.51 (0.17–1.23)	
**Q3**	90	89	0.34 (0.16–0.96)		0.36 (0.15–1.16)	
**Q4**	82	96	0.33 (0.14–0.79)		0.41 (0.17–1.08)	
**B12** ^**c**^ **(pg/mL)**				0.113		0.216
**Q1**	109	68	1.00 (reference)		1.00 (reference)	
**Q2**	93	77	0.66 (0.31–1.59)		0.77 (0.33–1.98)	
**Q3**	101	77	0.71 (0.31–1.73)		0.95 (0.37–2.35)	
**Q4**	74	95	0.49 (0.22–1.17)		0.58 (0.24–1.43)	

*CI, confidence interval; Q, Quintile; D, dementia; MCI, mild cognitive impairment; OR, odds ratio; SCD, subjective cognitive decline*.

*Linearity test: Pearsons correlation coefficient, r = 0.7; p = 0.027; coefficient of determination, R^2^ = 0.69*.

* *Fifth quintile is the reference group; Quintiles: Folic acid: Q1: 0.7–9.5 ng/dL; Q2: 9.6–18.4ng/dL; Q3: 18.5–27.3 ng/dL; Q4: 27.4–36.2 ng/dL; Q5: 36.3–45.1 ng/dL; Vitamin B12: Q1:43–210 pg/mL; Q2: 211–378 pg/mL; Q3: 379–546pg/mL; Q4: 547–714pg/mL; Q5: 715–879 pg/mL*.

a *p-values for linear trend; conditional logistic regression analysis*.

b *Adjusted model was adjusted for regular exercise by conditional multiple logistic regression analysis*.

c *Removed 13 outliers with serum vitamin B12 levels ≥ 2,000 pg/mL*.

## Discussion

To our knowledge, this is the first study that seeks to determine associations between endocrine and metabolic disorders and cognitive impairment in Peru, and one of the few in the LA region. In this cross-sectional study of 720 patients older than 60 years of age presenting to a specialized center in Lima for an initial evaluation of cognitive complaints, we found that patients with a diagnosis of dementia had lower folate levels and patients with MCI had higher fT3 levels, when compared with a group of patients with SCD but no objective evidence of dementia. Importantly, the mean and standard deviations of thyroid hormone levels, vitamin B12 and folate levels were within the normal range in all three groups. Despite this, we found that those in the lowest TSH quintiles had a dementia risk of nearly 3 times when compared with the SCD group in unadjusted models. However, we found no statistically significant associations between cognitive impairment and thyroid dysfunction, serum B12 or folic acid deficiencies after controlling for relevant covariates. We did, however, find that vitamin B12 deficiency and indeterminate levels of vitamin B12 were more prevalent in the dementia group, however, the causation of dementia cannot be determined based on these analyses. Thus, some of our findings do not strongly support the notion that these metabolic or endocrine disorders are important independent contributors to cognitive impairment in older Peruvians from Lima. However, our finding those with the lowest TSH levels had a greater dementia risk compared with the SCD group suggests a possible association between hyperthyroidism and cognitive impairment in our population.

Thyroid dysfunction is of particular importance given iodine deficiency is prevalent in many low- and middle-income countries, and can lead to thyroid dysfunction. In Latin America and the Caribbean, 10% of the general population of adults and children have insufficient iodine intake, and in Peru 11.8% (95% CI: 10.9–12.7%) have iodine deficiency (53) increasing their risk of developing a thyroid disorder. The prevalence of thyroid dysfunction among older adults from different regions of LA and its relationship to cognitive function is largely unknown. Similar to findings in our study, one study of Brazilians younger than 65 years of age found no relationship between subclinical thyroid status and cognitive function, but lower TSH was statistically associated with worse performance on executive function tests in extensively adjusted analyses (25). Another cross-sectional study of Brazilians older than age 65 noted a relationship between subclinical hyperthyroidism and all-cause dementia, but these analyses were only adjusted for age, not for other confounding factors such as the demographic variables that were accounted for in our study (age, sex, educational attainment, and regular exercise) (26). Similar to findings from these studies, our unadjusted results support the notion that lower TSH levels are associated with a greater risk of cognitive impairment, but after adjusting for covariates this association was weaker. Therefore, our study findings suggest that among older Peruvians from Lima with cognitive complaints, thyroid dysfunction does not represent a strong risk factor for cognitive impairment and other factors such as age or cardiovascular health may be more important contributors., however, further research is needed to determine the pathophysiological mechanisms underlying existing associations between cognitive impairment and thyroid dysfunction that were not uncovered in the present study.

Our study found that vitamin B12 deficiency was more common in the dementia group, however, there was no association between vitamin B12 deficiency and cognitive impairment (MCI + dementia combined) in unadjusted and adjusted multivariable analyses. More than 20% of the study population and nearly half of the dementia group was vitamin B12 deficient. The prevalence of vitamin B12 deficiency found in our study is high, but similar to that reported in another study on women with pre-eclampsia in Peru with 19% of the cohort having vitamin B12 deficiency (considered in that study to be a level <178.5 pg/mol) (54) and it was 9% in one international study of people with HIV in low- and middle-income countries (55). Several reasons for the high prevalence of B12 deficiency in our group, particularly in the dementia group, are possible. People with dementia have poor nutritional status prevalent in Latin America (56), which may be due to less meat ingestion (due to high costs, difficulty with chewing due to poor or no dentition) and lower absorption of vitamin B12 in older age (57), likely leading to a higher prevalence of B12 deficiency in our study population.

Although the findings of our study found no significant correlation between vitamin B12 deficiency and cognitive test scores in the dementia group after adjustment for relevant covariates, vitamin B12 deficiency was more common in the dementia group compared with the MCI and SCD. Other studies conducted in LA have found associations between vitamin B12 and folate levels and cognitive dysfunction in both unadjusted and adjusted analyses. In LA, B12 deficiency has been reported to be common with 17.4% (95%CI: 13.4–21.4%) of elderly Brazilians having B12 deficiency (58). Two studies, both from Brazil, have investigated the relationship between B12 and cognition and found that subjects with lower vitamin B12 levels have a greater risk of cognitive decline (59) and perform poorly on executive function tests in adjusted analyses (60). These studies were performed in a population of adults older than age 80, hence, our study population may have been too young to detect this association. In a study from Chile, investigators found that the risk of cognitive impairment increased with an increase in serum folate, but only in the setting of low vitamin B12 levels (61). The findings of our study cannot determine whether causation (whether vitamin B12 deficiency may have preceded onset of dementia, or whether B12 deficiency may lead to worse cognitive impairment in people with existing dementia), thus, further longitudinal work investigating causation is required.

In our study, serum folate concentrations were inversely associated with cognitive impairment in crude analyses, but not after additional adjustment. The relationship between folate levels and cognitive impairment in LA has also mostly been studied in Brazil, where lower serum folate levels were found among subjects with dementia of the AD type compared to MCI and controls in adjusted analyses (62). In our study, folate levels were lower in the dementia group compared with a group of patients with SCD. It is possible that persons with dementia could develop poor nutritional habits leading to vitamin B12 and folate deficiencies, but in our study the majority of participants had normal folate levels suggesting adequate micronutrient nutritional intake. Moreover, it is important to note that mandatory folate fortification in wheat flour is now standard practice in Peru (63), which likely accounts for the low prevalence (1.7%) of folate deficiency in the population studied. In addition, our study took place in a private neurology clinic in Lima, Peru, which likely represents patients of higher socioeconomic status with lower risk of nutritional deficiencies leading to vitamin deficiencies. However, despite lower folate levels in the dementia group in our study, there was no association between MMSE nor IFS scores with folate levels.

Possible explanations for a link between folate deficiency and cognitive dysfunction include impaired methylation reactions in the brain and insufficient methyl groups that are required for the synthesis of myelin, neurotransmitters, and membrane phospholipids (64). Deficiencies of cofactors involved in methionine and homocysteine metabolism (such as folate, vitamin B12, and vitamin B6) can result in hyperhomocysteinemia, and folate plays a crucial role in the methionine-homocysteine cycle (65). Several studies demonstrate that folate supplementation can affect cognitive function by diminishing serum homocysteine levels (66). Low homocysteine is also an independent risk factor for altered endothelial and hemostatic function (67). Due to lack of availability of homocysteine levels in routine clinical practice in Peru, we did not measure homocysteine levels, which may have mediated the lack of effect of vitamin B12 deficiency on dementia risk (11, 20). For example, a study from China found that elevated serum homocysteine levels were associated with increased AD risk, but higher vitamin B12 and folate levels were protective factors (20). Therefore, vitamin B12 and folate are not sufficient on their own to explain a possible etiology of reversible dementias, highlighting the importance of measuring these other levels in parallel with vitamin B12 and folate levels.

Our study has limitations. First, this is a cross-sectional retrospective study preventing the establishment of causal relationships and temporal associations. A prospective, longitudinal study with multiple measurements of serum metabolic levels over time would allow for correlation with neurocognitive changes, particularly with treatment initiation of a newly-detected metabolic disorder. Secondly, we excluded participants who were receiving treatment for a known previously diagnosed thyroid disorder or B12 or folate deficiency; thus, our results may not be generalizable to those with a known metabolic disorder already receiving treatment at baseline. In addition, we did not measure homocysteine or methylmalonic acid levels which may be the mediators of vitamin B12 deficiency in certain cases. Next, this was a convenience sample of Spanish-speaking generally healthy older adults attending a multi-disciplinary specialized neurological center in an urban setting, thus, limiting the external validity and generalizability of these study results to the general population of Peru, particularly rural communities that may experience a higher prevalence of nutritional deficiencies and may not have access to a specialized clinic. In addition, our population is not representative of the entire Peruvian population as many patients in our cohort had at least a high school or secondary school level education, excluding populations of lower education and non-native Spanish speakers. Next, because the screening phase of our study used brief cognitive screening tools whose scores may be influenced by factors such as educational level, there may have been initial group misclassification of the SCD group. Moreover, the SCD group was a group of persons with memory complaints but with normal results on cognitive testing, which may have been explained by existing subclinical metabolic disorders that may not have manifested as a clinical cognitive disorder. However, group assignment into dementia or MCI was completed in a systematic fashion using results from the complete neuropsychological test battery, and any group disagreement was resolved by consensus between the study team. Despite these limitations, we present the first study on metabolic disorders and cognitive impairment conducted in a well-characterized population with a large sample size (*N* = 720) of older Peruvians using a standardized evaluation protocol including patients with both dementia and MCI.

## Conclusions

Our results indicate that metabolic and endocrine disorders (thyroid dysfunction, B12, and folic acid deficiency) are not associated with dementia or MCI cross-sectionally in a population of older adults with cognitive complaints from Lima, Peru. Our study results add to existing evidence from other regions of LA which indicate that these disorders may not be significantly associated with cognitive impairment in the region despite a high prevalence of vitamin B12 deficiency found in our study. We found that B12 deficiency or borderline deficiency was present in more than one-third of those with dementia in our study population, however, no clear association was found between vitamin B12 levels and neuropsychological test results. Our study results may demonstrate that cognitive symptoms were likely a result of neurodegenerative disorders (such as AD, for example) and that potentially reversible metabolic and endocrine causes may be an incidental comorbidity. Because our study is cross-sectional, we are unable to determine if thyroid dysfunction, B12 or folate deficiencies are incidental or may relate directly to cognitive decline. Our study has emphasized that without longitudinal measurements of metabolic alterations and correlation with cognitive decline over time, it remains important to check thyroid function, vitamin B12, and folate levels during first-time consultation for cognitive impairment. Future work may investigate metabolic dysfunction longitudinally and its role in cognitive decline over time, particularly in LA where a greater burden of nutritional deficiencies may lead to metabolic disorders.

## Data Availability Statement

The raw data supporting the conclusions of this article will be made available by the authors, without undue reservation.

## Ethics Statement

The studies involving human participants were reviewed and approved by Hospital Nacional Docente Madre Niño San Bartolomé. The patients/participants provided their written informed consent to participate in this study.

## Author Contributions

MD: study concept and design, analysis and interpretation, and drafting of manuscript. NC: study concept and design, drafting of manuscript, and critical revision of the manuscript for important intellectual content. RM and DL: study concept and design, drafting of manuscript, critical revision of the manuscript for important intellectual content, and major role in acquisition of data. EH-P: statistical analyses and interpretation of results, drafting of manuscript. MP-C, JC-A, and CG: critical revision of the manuscript for important intellectual content, major role in acquisition of data. SL: study concept and design, critical revision of the manuscript for important intellectual content. All authors contributed to the article and approved the submitted version.

## Conflict of Interest

The authors declare that the research was conducted in the absence of any commercial or financial relationships that could be construed as a potential conflict of interest.

## Publisher's Note

All claims expressed in this article are solely those of the authors and do not necessarily represent those of their affiliated organizations, or those of the publisher, the editors and the reviewers. Any product that may be evaluated in this article, or claim that may be made by its manufacturer, is not guaranteed or endorsed by the publisher.
