# Supplementary material for: Thyroid Dysfunction, Vitamin B12, and Folic Acid Deficiencies Are Not Associated With Cognitive Impairment in Older Adults in Lima, Peru
Source: Front Public Health. 2021 Sep 6;9:676518. doi: 10.3389/fpubh.2021.676518 (PMC8450418; doi:10.3389/fpubh.2021.676518)
Supplement: Supplementary file 1 [file Data_Sheet_1.PDF]

Supplementary Material I: Mini-Mental State Exam, Peruvian Spanish version

**Cada respuesta correcta vale 1 punto**		PUNTAJE	
		ACTUAL	IDEAL
**Orientacion en el tiempo**	¿Qué fecha es hoy? (por ej.25+/-1día es correcto)		1
(No induzca las respuestas)	¿Qué día de la semana es hoy?(por ej. Lunes)		1
	¿En qué mes estamos? (por ej. Julio)		1
	¿En qué estación del año estamos? (por ej. Invierno)		1
	¿En qué año estamos?		1
**Orientación en el espacio**	¿Dónde estamos? (por ej. un hospital, con nombre propio)		1
(No induzca las respuestas)	¿En qué direccion estamos?		1
	¿En qué ciudad estamos?		1
	¿En qué departamento/region estamos?		1
	¿En qué país estamos?		1
**Registro**	Pelota		1
(Enuncie las tres palabras clara y lentamente a un ritmo de una por segundo.	Bandera		1
Luego de haberlas dicho solicite a su paciente que las repita. La primera repetición determina el puntaje, pero haga que el paciente siga repitiendo hasta que aprenda las tres, hasta seis intentos.)	Árbol		1
**Atención y Cálculo**	Deletrear MUNDO al revés		
(Hágale deletrear MUNDO de atrás hacia delante -ODNUM-. Cada letra en el orden correcto vale 1 punto; o bien, pídale al paciente que realice restas consecutivas de a 7 unidades comenzando desde 100-93 86, 79, 72, 65-.Deténganse	(1 pto.por cada letra correcta: ODNUM) o bien, ¿Cuánto es 100-7?. (Hacer 5 restas (93) (86), (79), (72),(65):1 pto. Por cada respuesta correcta)		5

luego de 5 restas. Cada resta correcta vale 1 punto).			
**Evocación**<br>(Pregunte al paciente si puede recordar las tres palabras que antes repitió.)	Digame las tres palabras que antes repitió		3
**Lenguaje**			
**Nominación**<br>(Sólo un intento que vale 1 pto. para cada item.)	¿Qué es esto? (mostrarle un lápiz)<br>¿Qué es esto? (mostrarle un reloj)		1<br>1
**Repetición**<br>(Sólo un intento que vale 1 pto.)	Dígale a su paciente la siguiente frase: "La mazamorra tiene duraznos y guindones"		1
**Orden de tres comandos**<br>(Cada parte correctamente ejecutada vale 1 pto.)	Déle el papel a su paciente y dígale: "Tome este papel con la mano izquierda, dóblelo por la mitad y devuelvamelo con la mano derecha"		3
**Lectura**<br>(Otorgue 1 pto.sólo si el paciente cierra sus ojos)	Muéstrele la hoja que dice: " Cierre los ojos" y dígale: "Haga lo que aquí se indica, sin leerlo en voz alta".		1
**Escritura**<br>(No dicte la oración; ésta debe ser espontánea.Debe contener un sujeto y un vebo.No es necesario que la gramática y la puntuación sean correctas).	Déle a su paciente una hoja en blanco y pídale que escriba una frase.		1
**Copia**<br>(Para ser correcto, deben estar	Déle a su paciente la hoja que dice: "Copie esta figura", y pídale que copie		1

presentes todos los ángulos,los lados y las intersecciones.No se toman en cuenta temblor, líneas desparejas o no rectas. La distribución de las figuras copiadas debe ser igual al original.)	el dibujo.		
**Total MMSE:**			30
