# Supplementary material for: Thyroid Dysfunction, Vitamin B12, and Folic Acid Deficiencies Are Not Associated With Cognitive Impairment in Older Adults in Lima, Peru
Source: Front Public Health. 2021 Sep 6;9:676518. doi: 10.3389/fpubh.2021.676518 (PMC8450418; doi:10.3389/fpubh.2021.676518)
Supplement: Supplementary file 2 [file Data_Sheet_2.PDF]

INECO FRONTAL SCREENING [IFS]

SERIES MOTORAS	/3
**"Mire atentamente lo que estoy haciendo".** El examinador realiza tres veces la serie de Luria "puño, canto, palma". Ahora haga lo mismo con su mano derecha, primero conmigo y después solo." El examinador realiza las series 3 veces con el paciente y luego le dice. "Ahora, hágalo usted solo". Puntuación: 6 series consecutivas solo: 3 / al menos 3 series consecutivas solo: 2 / El paciente falla en 1 pero logra 3 series consecutivas con el examinador: 1 / El paciente no logra 3 series consecutivas con el examinador : 0	

INSTRUCCIONES CONFLICTIVAS	/3
**"Golpee dos veces cuando yo golpeo una vez".** Para asegurarse que el paciente comprendió bien la consigna, realice una serie de 3 pruebas: 1-1-1**. "Golpee una vez cuando yo golpeo dos veces"**. Para asegurarse que el paciente comprendió bien la consigna, realice una serie de 3 pruebas: 2-2-2. El examinador realiza la siguiente serie: 1-1-2-1-2-2-2-1-1-2. Puntuación: Sin error: 3 / Uno o dos errores: 2 / Mas de dos errores: 1 / El paciente golpea como el examinador al menos 4 veces consecutivas : 0	

CONTROL INHIBITORIO MOTOR	/3
**"Golpee una vez cuando yo golpeo una vez".** Para asegurarse que el paciente comprendió bien la consigna, realice una serie de 3 pruebas: 1-1-1. **"No golpee cuando yo golpeo dos veces".** Para asegurarse que el paciente comprendió bien la consigna, realice una serie de 3 pruebas: 2-2-2. El examinador realiza la siguiente serie: 1-1-2-1-2-2-2-1-1-2. Puntuación: Sin error: 3 / Uno o dos errores: 2 / Mas de dos errores: 1 / El paciente golpea como el examinador al menos 4 veces consecutivas : 0	

REPETICIÓN DE DÍGITOS ATRÁS			/6
2-4		5-7	
6-2-9		4-1-5	
3-2-7-9		4-9-6-8	
1-5-2-8-6		6-1-8-4-3	
5-3-9-4-1-8		7-2-4-8-5-6	
8-1-2-9-3-6-5		4-7-3-9-1-2-8	
9-4-3-7-6-2-5-8		7-2-8-1-9-6-5-3	

MESES ATRÁS											/2
Diciembre		Noviembre		Octubre		Septiembre		Agosto		Julio	
Junio		Mayo		Abril		Marzo		Febrero		Enero	

MEMORIA DE TRABAJO VISUAL			/4
1-2		3-4-2-1	
2-4-3		1-4-2-3-4	
Señale los cubos en el orden indicado, el paciente deberá copiar esta secuencia de movimientos en orden inverso. Hágalo despacio; el paciente decide que mano prefiere utilizar.			

REFRANES		/3
**Perro que ladra, no muerde**	Los que hablan mucho, suelen hacer poco.	
**A mal tiempo, buena cara**	Tomar una actitud positiva frente a las adversidades	
**En casa de herrero, cuchillo de palo**	Carecer de algo cuando por tu profesión u oficio, no debiera faltarte	
Ejemplo: ½ punto. Explicación Correcta: 1 Punto.		

CONTROL INHIBITORIO VERBAL	6
**Iniciación**	
Me pongo los zapatos y me ato los ………	
Peleaban como perro y ………..	
**Inhibición**	
Juan compró caramelos en el …………	
Ojo por ojo y diente por …………….	
Lave la ropa con agua y …………	
Iniciación: Escuche cuidadosamente esta oración y cuando yo haya terminado de leerla, usted debe decirme lo más rápido posible, qué palabra podría completar la frase. Consignar respuesta. Inhibición. Diferente: 2/ Rel. Semántica: 1/Palabra exacta: 0 punto. Esta vez quiero que me diga una palabra que no tenga ningún sentido en el contexto de la oración, que no tenga ninguna relación con la misma. Por ejemplo: Daniel golpeó el clavo con el ...lluvia.	

Puntaje Total = **/ 30** **Índice de Memoria de Trabajo (Digitos atrás + corsi) = / 10**
