# Supplementary material for: Thyroid Dysfunction, Vitamin B12, and Folic Acid Deficiencies Are Not Associated With Cognitive Impairment in Older Adults in Lima, Peru
Source: Front Public Health. 2021 Sep 6;9:676518. doi: 10.3389/fpubh.2021.676518 (PMC8450418; doi:10.3389/fpubh.2021.676518)
Supplement: Supplementary file 3 [file Data_Sheet_3.PDF]

Cuestionario de Actividades Funcionales de Pfeffer

(Pfeffer Functional Activities Questionnaire-PFAQ)

INSTRUCTIVAS PARA APLICACIÓN:

Con ayuda del cuidador o familiar, pregunte en el orden establecido una a una cada actividad.

De acuerdo a cada respuesta, califique según el grado establecido del 0 al 3.

Actividad	0	1	2	3	Total
1. Maneja el/ella su propio dinero?					
2. Es él/ella capaz de comprar ropa, cosas de casa, comestibles, solo?					
3. Es él/ella capaz de calentar agua para el café o té y apagar la cocina?					
4. Es él/ella capaz de preparar una comida?					
5. ¿Está al corriente de las noticias de su vecindario, de su comunidad?					
6. Es él/ella capaz de poner atención y entender y discutir un programa de radio o TV, diario o revista?					
7. Es él/ella capaz de recordar compromisos, acontecimientos familiares, vacaciones?					
8. Es él/ella capaz de manejar sus propios medicamentos?					
9. Es él/ella capaz de pasear por el vecindario y encontrar el camino de vuelta a casa?					
10. Es él/ella capaz de saludar a sus amigos adecuadamente?					
11. Puede él/ella ser dejado solo en casa en forma segura?					
**PUNTAJE TOTAL**					

Calificación de la actividad:

0: Normal
1: Difícilmente, pero lo hace
2: Requiere ayuda

0: Nunca lo ha hecho, pero podría hacerlo ahora.
1: Nunca lo ha hecho, y tendría dificultad para hacerlo.
3: Dependiente (no lo hace, ni con ayuda)

PUNTO DE CORTE:

Resultados mayores a 6 puntos, revelan algún tipo de compromiso funcional.

*Basados en Custodio N, Garcia A, Montesinos R, Escobar J y Bendezu L. Prevalencia de demencia en una población urbana de Lima-Peru: Estudio puerta a puerta. *An Fac Med 2008;69(4):233-238.*
