# Supplementary material for: Thyroid Dysfunction, Vitamin B12, and Folic Acid Deficiencies Are Not Associated With Cognitive Impairment in Older Adults in Lima, Peru
Source: Front Public Health. 2021 Sep 6;9:676518. doi: 10.3389/fpubh.2021.676518 (PMC8450418; doi:10.3389/fpubh.2021.676518)
Supplement: Supplementary file 4 [file Table_1.DOCX]

**Supplementary Table IV: Pearson’s correlation analysis between serum thyroid hormone levels with age, BMI, level education and brief cognitive test in all groups.**

| **Demographic and cognitive features** | **SCD** | | | | | | **MCI** | | | | | | **Dementia** | | | | | |
| --- | --- | --- | --- | --- | --- | --- | --- | --- | --- | --- | --- | --- | --- | --- | --- | --- | --- | --- |
|  | **Free T3** | | **Free T4** | | **TSH** | | **Free T3** | | **Free T4** | | **TSH** | | **Free T3** | | **Free T4** | | **TSH** | |
|  | γ | **P** | γ | **P** | γ | **P** | γ | **P** | γ | **P** | γ | **P** | γ | **P** | γ | **P** | γ | **P** |
| **Age** | -0.15 | 0.094 | -0.11 | 0.086 | -0.16 | 0.126 | -0.10 | 0.231 | -0.14 | 0.145 | -0.18 | 0.218 | **-0.31** | **0.002** | -0.13 | 0.067 | -0.12 | 0.248 |
| **BMI** | 0.01 | 0.091 | 0.19 | 0.231 | 0.12 | 0.076 | -0.12 | 0.087 | 0.02 | 0.678 | -0.02 | 0.423 | -0.15 | 0.178 | -0.09 | 0.423 | - 0.13 | 0.432 |
| **Years of education** | -0.16 | 0.086 | **-0.35** | **0.031** | -0.03 | 0.079 | 0.21 | 0.082 | 0.02 | 0.786 | -0.14 | 0.063 | 0.03 | 0.832 | 0.13 | 0.087 | -0.15 | 0.128 |
| **MMSE** | 0.15 | 0.653 | 0.23 | 0.087 | 0.12 | 0.083 | -0.23 | 0.093 | -0.18 | 0.174 | -0.17 | 0.082 | -0.10 | 0.095 | -0.11 | 0.165 | **-0.21** | **0.02** |
| **IFS** | 0.09 | 0.076 | 0.12 | 0.541 | 0.12 | 0.432 | 0.07 | 0.076 | 0.11 | 0.456 | -0.15 | 0.061 | -0.20 | 0.071 | -0.12 | 0.423 | **-0.32** | **0.01** |

**Supplementary Table V: Pearson’s correlation analysis between vitamin B12 and folic acid levels with age, BMI, level education and brief cognitive test in all groups.**

| **Demographic and cognitive features** | **SCD** | | | | **MCI** | | | | **Dementia** | | | |
| --- | --- | --- | --- | --- | --- | --- | --- | --- | --- | --- | --- | --- |
|  | **Vitamin B12** | | **Folic acid** | | **Vitamin B12** | | **Folic acid** | | **Vitamin B12** | | **Folic acid** | |
|  | γ | P | γ | P | γ | P | γ | P | γ | P | γ | P |
| **Age** | -0.21 | 0.072 | 0.21 | 0.561 | -0.52 | 0.074 | 0.32 | 0.231 | **-0.35** | **0.023** | **-0.43** | **0.012** |
| **BMI** | 0.05 | 0.132 | 0.19 | 0.752 | 0.11 | 0.076 | 0.43 | 0.072 | 0.06 | 0.083 | 0.23 | 0.156 |
| **Years of education** | 0.18 | 0.067 | 0.16 | 0.921 | 0.33 | 0.069 | 0.51 | 0.346 | 0.29 | 0.232 | -0.21 | 0.082 |
| **MMSE** | 0.33 | 0.593 | -0.67 | 0.732 | 0.19 | 0.667 | 0.15 | 0.082 | 0.17 | 0.539 | -0.18 | 0.098 |
| **IFS** | 0.29 | 0.069 | -0.23 | 0.341 | 0.11 | 0.162 | 0.27 | 0.622 | 0.21 | 0.763 | -0.27 | 0.091 |

**Supplementary Table VI: Demographic, clinical and cognitive characteristics of participants with normal levels of vitamin B12, indeterminate vitamin B12 ranges and deficiency of vitamin B12*. Instituto Peruano de Neurociencias, 2014-2020.**

| **Characteristic** | **Vitamin B12 deficiency**  **(N=151)** | **Vitamin B12 indeterminate**  **(N=139)** | **Vitamin B12 normal**  **(N=430)** | **P value Vitamin B12 deficiency vs**  **Vitamin B12 normal** | **P value Vitamin B12 indeterminate vs**  **Vitamin B12 normal** | **P value Vitamin B12 deficiency vs**  **Vitamin B12 indeterminate** |
| --- | --- | --- | --- | --- | --- | --- |
| **SCD, n (%)** | 28 (18.5%) | 61 (43.9%) | 225 (52.3%) | **p<0.001** | p=0.084 | **p<0.001** |
| **MCI, n (%)** | 49 (32.5%) | 37 (26.6%) | 148 (34.4%) | 0.660 | 0.088 | 0.277 |
| **Dementia, n (%)** | 74 (49%) | 41 (29.5%) | 57 (13.3%) | **p<0.001** | p<0.001 | **0.001** |
| **Age, years (mean ± SD)** | 64.8 ± 7.2 | 64.3 ± 6.1 | 65.7 ± 4.9 | 0.223 | 0.179 | 0.762 |
| **Education, years (mean ± SD)** | 10.5 ± 3.2 | 10.7 ± 3.5 | 11.1 ± 2.5 | 0.558 | 0.458 | 0.673 |
| **Female, n (%)** | 78 (51.7%) | 73 (52.5%) | 223 (51.8%) | 0.892 | 0.632 | 0.643 |
| **BMI, kg/m2 (mean ± SD)** | 22.5 ± 3.1 | 21.9 ± 3.4 | 22.3 ± 2.6 | 0.712 | 0.456 | 0.351 |
| **MMSE score (mean ± SD)** | 21.5 ± 4.3 | 22.1 ± 3.1 | 21.3 ± 5.8 | 0.562 | 0.458 | 0.512 |
| **IFS score (mean ± SD)** | 22.9 ± 2.1 | 21.1± 3.5 | 21.2 ± 2.1 | 0.158 | 0.124 | 0.217 |

*Vitamin B12 deficiency (<80 pg/mL); indeterminate Vitamin B12 status (81-200 pg/mL); normal Vitamin B12 (>200 pg/mL).
